# Students’ motivation to study dentistry in Malaysia: an analysis using confirmatory factor analysis

**DOI:** 10.1186/s12960-015-0040-4

**Published:** 2015-06-12

**Authors:** Muhd Firdaus Che Musa, Eduardo Bernabé, Jennifer E Gallagher

**Affiliations:** Division of Population and Patient Health, King’s College London Dental Institute at Guy’s, King’s College and St Thomas’ Hospitals, Denmark Hill Campus, Bessemer Road, London, SE5 9RS UK

**Keywords:** Motivation, Professional career, Dental students, Malaysian, Dental education

## Abstract

**Introduction:**

Malaysia has experienced a significant expansion of dental schools over the past decade. Research into students’ motivation may inform recruitment and retention of the future dental workforce. The objectives of this study were to explore students’ motivation to study dentistry and whether that motivation varied by students’ and school characteristics.

**Methods:**

All 530 final-year students in 11 dental schools (6 public and 5 private) in Malaysia were invited to participate at the end of 2013. The self-administered questionnaire, developed at King’s College London, collected information on students’ motivation to study dentistry and demographic background. Responses on students’ motivation were collected using five-point ordinal scales. Confirmatory factor analysis (CFA) was used to evaluate the underlying structure of students’ motivation to study dentistry. Multivariate analysis of variance (MANOVA) was used to compare factor scores for overall motivation and sub-domains by students’ and school characteristics.

**Results:**

Three hundred and fifty-six final-year students in eight schools (all public and two private) participated in the survey, representing an 83% response rate for these schools and 67% of all final-year students nationally. The majority of participants were 24 years old (47%), female (70%), Malay (56%) and from middle-income families (41%) and public schools (78%). CFA supported a model with five first-order factors (professional job, healthcare and people, academic, careers advising and family and friends) which were linked to a single second-order factor representing overall students’ motivation. Academic factors and healthcare and people had the highest standardized factor loadings (0.90 and 0.71, respectively), suggesting they were the main motivation to study dentistry. MANOVA showed that students from private schools had higher scores for healthcare and people than those in public schools whereas Malay students had lower scores for family and friends than those from minority ethnic groups. No differences were found by age, sex, family income and school type.

**Conclusion:**

Using CFA, this study shows that academic factors were the main motivation to study dentistry in this group of Malaysian students. There were also variations in students’ motivation by students’ ethnicity and school sector but not by other factors.

## Introduction

There is a growing recognition that a motivated and skilled workforce is necessary to deliver an optimal health service to the population [1]. Research into students’ motivation and career expectations may assist dental educators and health providers to develop better models to recruit and retain the workforce for the benefit of both the community and profession [2,3].

Several conceptual models and theories have been proposed to explain vocation and career decision-making [4-6], of which Holland’s typology of vocational personalities and work environments [5], is arguably the most influential [4]. However, the literature on students’ motivation to choose dentistry as a professional career has been quite empirical, deriving influencing factors from data reduction techniques such as exploratory factor analysis [7-9]. Gallagher et al. [10] developed an instrument, informed by qualitative research, assessing five domains underlying motivation to study dentistry (professional job, healthcare and people, academic, careers advising and family and friends).

Most studies on motivation to study dentistry come from developed countries [10-20]. These studies suggest that features of the job [10-12] and desire to work with people/altruism [14,15,17,20], are the primary motivation to study dentistry. One questionnaire survey of individual schools in 13 countries covering 6 continents claimed that having enough time for the family and altruism were the main motivation to study dentistry; however, the first-choice influence varied between countries [2]. Another study across Western and Eastern countries found that most dental students from both regions shared similar concerns for personal, altruism and academic interest and suggested that differences may relate to their future career options [20]. However, there is little evidence on whether students in developing countries have the same motivation to study dentistry.

In relation to the factors related to motivation to study dentistry as a career, there is evidence of differences by age [21], sex [7,10,21], ethnicity [10,22,23], and mode of entry [10]. It is, however, unknown whether school characteristics may have an impact on students’ motivation [16]. The majority of these studies have been conducted in either state/public [21,24,25], or private schools [26] – often merely single schools – and there are no cross-sector studies at the national level.

The present study uses Malaysia as a case study for a middle-income country. Malaysia has seen a rapid expansion in dental schools with 12 new schools opened in the past decade, and an average of a thousand graduates annually. Understanding students’ views may contribute to identify challenges and possible solutions so that the future workforce is recruited and retained to address population needs. Caries levels remain high in Malaysia despite recent declines among 6- (from a dft [the average number of decayed and filled primary teeth] of 4.1 in 1997 to 3.6 in 2007) [27] and 12-year-olds (from a DMFT [the average number of decayed, missing and filled permanent teeth] of 3.3 in 1997 to 2.1 in 2007) [28]. In addition, 89% and 34% of 35- to 44-year-old adults had dental caries and periodontal disease in 2010 [29]. As for dental workforce, Malaysia has dentists (generalists and specialists) and dental auxiliaries (Malaysian dental nurses, dental technicians and dental surgery assistants). In 2011, there were 4289 dentists and a dentist-to-population ratio of 1 to 6810 [30,31]. Although this figure is close to the government’s target of having 1 dentist to 4000 population by 2020 [32], the distribution of dentists varies by state, with most working in the Peninsula of Malaysia and in urban areas [33,34].

The objectives of this study were to explore students’ motivation to study dentistry as a professional career and to examine whether their motivation to study dentistry varies by students’ and school characteristics.

## Methods

### Study population

A cross-sectional survey was carried out among final-year dental students registered for the academic year 2013/2014 in the 11 dental schools in Malaysia (6 public and 5 private). All 530 students in those dental schools were invited to participate in this study. The study protocol was approved by KCL Biomedical Sciences, Dentistry, Medicine and Natural and Mathematical Sciences Research Ethics Subcommittee (Reference BDM/12/13-129). Completion and return of the questionnaire (either partially or completely) was taken as consent to participate as outlined in the information sheet provided to students.

### Data collection

Data were collected using a self-administered questionnaire. Approach letters were sent to all dental schools informing their authorities about the purpose of the study and requesting permission to conduct the survey. After approval, students were given the participant’s information sheet by the institution and have at least 24 h to consider taking part in this study. On the day of appointment, the students received a verbal explanation by a researcher. The questionnaire which required up to 30 min for completion was administered at a convenient time for the institution and its students, within a classroom setting (during lunch time or after lectures). The questionnaire was administered in English as this is the medium of teaching in all dental schools in Malaysia.

The original English questionnaire (Gallagher Motivation Instrument, GMI; [10]) was informed by qualitative research among final-year dental students and vocational dental practitioners (new graduates in the UK) [12,35]. It has been used with final-year dental students in the UK [10,13] and abroad [21]. The questionnaire has 31 questions in 6 sections. It explores student’s vision of dentistry as a professional career (three questions), short-term career aspirations (five), long-term career aspirations (six), influences on their career choices (five), views on state healthcare (three) and their background information (age, gender, ethnicity, family income and social background) (eight). Responses on motivation to study dentistry were collated (under students’ vision of dentistry) using five-point ordinal scales, ranging from very important (coded as 5) to not important at all (coded as 1) across 23 motivation items. There was an open-ended question provided at the end of the section to enable collection of additional relevant information.

In the background section, modified for the Malaysian context, students identified themselves as Malay, Chinese, Indian, Kadazan, Dayak, Sikh and other ethnicities; responses were later grouped as Malay, Chinese and others for analysis. Monthly household income was collected using a question from the national Household Income Survey and categorized as low (Malaysian Ringgit < 2000), middle (RM 2001–5000) or high (RM > 5000). Two indicators were used to characterize dental schools: the first was school sector classified as public or private and the second was school type, classified as established (more than 10 years of operation) and new (less than 10 years of operation).

The GMI questionnaire was revised to improve its cross-cultural and face validity. First, questions on socio-demographic characteristics and health services were amended to make them suitable to the Malaysian context. A pilot study was conducted with 20 final-year dental students at 2 universities. These schools represented the full range of ethnic groups and student backgrounds. The questionnaire was revised in line with students’ recommendations for improvement. The internal consistency of the questionnaire was acceptable, with a Cronbach’s alpha of 0.85.

### Statistical analysis

**Figure 1:**
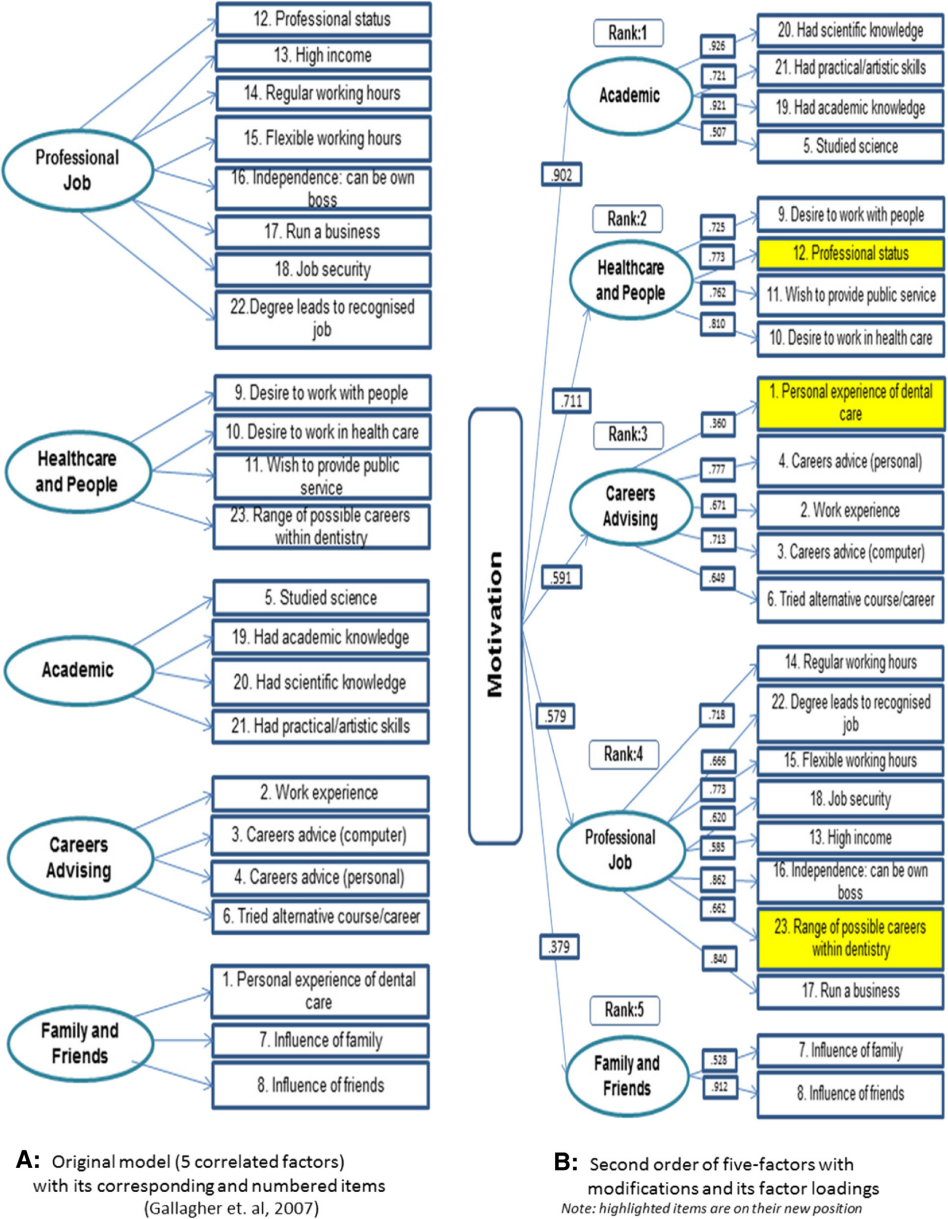
Comparison by its corresponding items across two models; original model **(A)** and modification model **(B)**.

Descriptive analysis was first undertaken to summarize the sample characteristics and participants’ responses to all 23 motivation items. Subsequent analyses were conducted in M*plus* version 6.1 using full-information maximum likelihood estimation under the assumption that items were missing at random to handle item non-response [36]. In this sample, only 19 (5%) participants had 1 or more missing items. As item responses were collected using ordinal scales, the weighted least square method was used to estimate model parameters [37]. We tested two alternatives factor structures for students’ motivation to study dentistry using confirmatory factor analysis (CFA). First, we fitted a five-correlated factor model (Figure 1A) derived from a previous study [10]. Second, we tested a second-order model that comprised five first-order factors each combined with a single second-order factor (Figure 1B). The eight items on professional job, four on healthcare and people, four on academic, five on careers advising and two on family and friends were assigned to each of the first-order factors which, in turn, were linked to the second-order factor describing overall motivation. The second model differs from the first by implicitly testing that the five first-order factors were related to each other because they were all underlying measures of students’ motivation to study dentistry.

The goodness-of-fit of each model was tested using the chi-square test. As this test is highly sensitive to a large-sample size [38], the comparative fit index (CFI) and the root mean square error of approximation (RMSEA) were also used. CFI values above 0.90 and RMSEA values below 0.10 were indicative of good fit to the data [38,39]. If neither of the two models had good fit to the data, modification indices supported by theoretical arguments were used to improve model fit.

Factor scores (which have a mean of zero and standard deviation of one) were generated from the model with the best goodness-of-fit statistics. We used the multivariate analysis of variance (MANOVA) to compare, first jointly and then individually, the five domain scores and the overall motivation score by participants’ socio-demographic and school characteristics. MANOVA allows comparing multiple and inter-correlated outcome measures (six in this study, namely the five domain and the total latent scores), compensating for multiple comparisons by using omnibus tests for multiple outcomes and multiple groups, controlling for confounders and testing for interactions between explanatory variables. *Post hoc* comparisons between pairs of groups were conducted using Scheffe’s test and only when omnibus tests were statistically significant.

## Results

**Figure 2:**
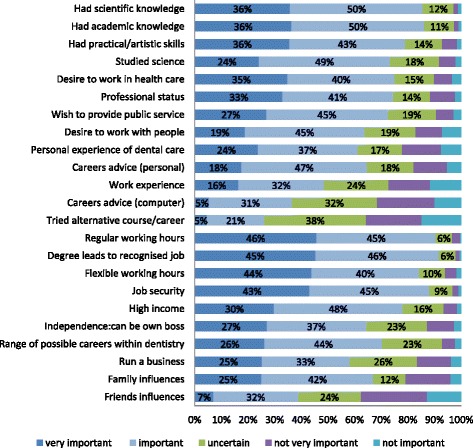
Students’ responses to Gallagher Motivation Instrument: 23 items describing specific motivations to study dentistry.

Eight out of the 11 dental schools in Malaysia agreed to participate (all 6 public plus 2 private schools). Of the 431 final-year students across the 8 schools, 356 (83%) completed the questionnaire and were included in this analysis. This represented 67% of the national population of final-year students registered in the 11 dental schools for 2013/14. The characteristics of the sample are shown in Table 1. Regular working hours (91%), degree leads to recognized job (91%) and job security (88%), on one hand, and academic knowledge (86%) and scientific knowledge (86%), on the other, were the items with the highest proportion of very important/important responses (Figure 2).

**Table 1:** **Characteristic of final-year Malaysian dental student Respondents, 2013/14 (** ***n*** **= 356)**

**Characteristics**	**Groups**	***n***	**%**
Sex	Men	103	28.9
	Women	253	71.1
Age group	<24 years	134	37.6
	24 years	169	47.5
	>24 years	53	14.9
Ethnicity	Malay	195	54.8
	Chinese	129	36.2
	Others	32	9.0
Family income	Low	88	24.7
	Middle	136	38.2
	High	109	30.6
	Missing	23	6.5
School sector	Government	273	76.7
	Private	83	23.3
School type	New	197	55.3
	Established	159	44.7

Note: response rate of 83% across eight responding dental schools.

CFA showed that the original correlated five-factor model had a poor fit to the data (chi^2^: 574.1, degrees of freedom: 61, CFI: 0.823, RMSEA: 0.154). The second-order factor model with a higher latent factor representing motivation did not have a good fit to the data either (chi^2^: 1016.4, degrees of freedom (df): 225, CFI: 0.740; RMSEA: 0.099). Three modifications were implemented to improve model fit. They required moving, in that order, item 23 (*range of possible career options*) from the factor healthcare and people to the factor professional job, item 12 (*professional status*) from the factor professional job to the factor healthcare and people and item 1 (*personal experience of dental care*) from the factor family and friends to the factor careers advising. The revised second-order factor model had good fit to the data (chi^2^: 957.0, df: 225, CFI: 0.901, RMSEA: 0.096). Factor loadings for the five latent motivation domains ranged from 0.902 for academic to 0.379 for family and friends (Figure 1B).

Latent factor scores representing overall motivation and the five subdomain scores were compared by students’ and school characteristics (Table 2). There were significant differences in the factor scores for healthcare and people by school sector and in the factor score for family and friends by ethnicity. More specifically, students from private schools had higher scores for healthcare and people compared to those in public schools whereas Malay students had lower scores for family and friends than students from ethnic minority groups. There were no significant two-way interactions between students’ and school characteristics when they were added to the main effects model (all *P* < 0.05).

**Table 2:** **Comparison of domain and overall scores for motivation by students’ and school characteristics (** ***n*** **= 356)**

**Characteristics**		**Professional job**	**Healthcare and people**	**Academic**	**Careers advising**	**Family and friends**	**Overall motivation**
		**Mean (95% CI)**	**Mean (95% CI)**	**Mean (95% CI)**	**Mean (95% CI)**	**Mean (95% CI)**	**Mean (95% CI)**
*All Participants*		0.03 (−0.07, 0.12)	0.10 (−0.02, 0.22)	0.05 (−0.04, 0.14)	0.03 (−0.03, 0.09)	0.04 (−0.05, 0.12)	0.04 (−0.02, 0.09)
Sex	Men	0.03 (−0.09, 0.16)	0.16 (0.01, 0.31)	0.12 (0.01, 0.23)	0.07 (−0.01, 0.14)	0.07 (−0.04, 0.17)	0.08 (0.01, 0.15)
	Women	0.03 (−0.09, 0.16)	0.04 (−0.09, 0.16)	−0.01 (−0.11, 0.08)	0.00 (−0.06, 0.07)	0.01 (−0.08, 0.10)	0.00 (−0.06, 0.06)
Age group	<24 years	0.02 (−0.10, 0.14)	0.15 (0.01, 0.29)	0.07 (−0.03, 0.17)	0.06 (−0.02, 0.13)	0.04 (−0.06, 0.14)	0.05 (−0.01, 0.12)
	24 years	−0.01 (−0.12, 0.10)	0.09 (−0.04, 0.23)	0.02 (−0.07, 0.12)	0.03 (−0.03, 0.10)	0.03 (−0.06, 0.13)	0.02 (−0.04, 0.08)
	>24 years	0.06 (−0.10, 0.23)	0.05 (−0.15, 0.25)	0.06 (−0.08, 0.20)	0.01 (−0.09, 0.11)	0.04 (−0.10, 0.18)	0.04 (−0.06, 0.13)
Ethnicity	Malay	−0.03 (−0.17, 0.11)	0.08 (−0.09, 0.25)	0.07 (−0.06, 0.19)	0.06 (−0.03, 0.14)	−0.08 (−0.20, 0.04)*	0.04 (−0.04, 0.11)
	Chinese	−0.03 (−0.13, 0.08)	0.11 (−0.02, 0.24)	0.03 (−0.06, 0.13)	−0.02 (−0.09, 0.04)	0.04 (−0.05, 0.13)	0.02 (−0.04, 0.08)
	Others	0.14 (−0.05, 0.32)	0.10 (−0.13, 0.33)	0.05 (−0.11, 0.22)	0.06 (−0.05, 0.18)	0.15 (−0.01, 0.32)*	0.05 (−0.05, 0.16)
Family income	Low	0.15 (0.02, 0.28)	0.21 (0.05, 0.36)	0.13 (0.02, 0.24)	0.04 (−0.04, 0.12)	0.07 (−0.05, 0.18)	0.09 (0.02, 0.16)
	Middle	0.04 (−0.07, 0.16)	0.08 (−0.06, 0.22)	0.06 (−0.04, 0.17)	0.02 (−0.05, 0.09)	0.02 (−0.08, 0.11)	0.04 (−0.02, 0.10)
	High	−0.06 (−0.18, 0.06)	0.10 (−0.05, 0.25)	0.09 (−0.02, 0.20)	0.04 (−0.04, 0.11)	0.06 (−0.05, 0.16)	0.05 (−0.02, 0.12)
	Not stated	−0.04 (−0.26, 0.19)	0.00 (−0.28, 0.28)	−0.08 (−0.28, 0.12)	0.04 (−0.10, 0.17)	0.01 (−0.18, 0.21)	−0.03 (−0.16, 0.10)
School sector	Public	0.09 (−0.02, 0.20)	−0.03 (−0.17, 0.10)*	0.00 (−0.10, 0.10)	0.01 (−0.06, 0.08)	0.09 (−0.01, 0.18)	0.01 (−0.06, 0.07)
	Private	−0.04 (−0.20, 0.13)	0.23 (0.03, 0.43)*	0.10 (−0.04, 0.25)	0.06 (−0.05, 0.16)	−0.01 (−0.15, 0.13)	0.07 (−0.02, 0.16)
School type	New	−0.03 (−0.13, 0.07)	−0.01 (−0.13, 0.12)	−0.01 (−0.10, 0.08)	0.00 (−0.06, 0.06)	0.03 (−0.05, 0.12)	−0.01 (−0.06, 0.05)
	Established	0.08 (−0.06, 0.22)	0.20 (0.03, 0.37)	0.11 (−0.01, 0.24)	0.07 (−0.02, 0.15)	0.04 (−0.07, 0.16)	0.08 (−0.00, 0.22)

All comparisons were made using multivariate analysis of variance (MANOVA). Significant differences are indicated with asterisks.

## Discussion

Academic factors were the primary motivation to study dentistry in these Malaysian students, followed by healthcare and people, careers advising, professional job and family and friends. We also found differences in students’ motivation by school sector (public/private) and ethnicity. No other differences were found by students’ or school characteristics.

Some limitations of this study need to be addressed. First, we had a lower participation from private institutions, and therefore, comparisons between the public and private sector need to be treated with caution. Second, the present findings relate to current final-year students and may not be generalizable to students in other years of study or final-year students over time. However, the demographic profile of participants was relatively close to the population of existing dental practitioners in 2013 (65% women; 51% Malay, 33% Chinese and 16% from other groups) [33]. Third, some may argue that career choice should be evaluated among dental applicants or first-year students rather than final-year students [7,17]. However, there is evidence suggesting that students’ motivation remains fairly stable over the course of the dental programme [11].

CFA showed that neither of the two hypothesized models had good fit to the data. Three amendments were implemented based on theoretical grounds and modification indices to improve model fit. Two of them were straightforward, moving item 23 from healthcare and people to professional job and item 1 from family and friends to careers advising. The third modification, moving item 12 (*professional* status) from professional job to healthcare and people was more of a challenge. However, it was done on the grounds that unlike other countries, where the professional status of dentistry is commonly attached to economic or financial standing, the professional standing of dentists in Malaysia comes from being healers [2] and the respect and prestige associated with such a role in society [40].

The first important finding of this study was that Malaysian students were primarily motivated to study dentistry by academic factors, which contrasts sharply with evidence from developed countries where professional features of the job are more important for students [2,10,13,17,21]. Our findings challenge the view that dental students across the world are financially motivated [26]. Malaysia is a country placing a great emphasis on scientific and technological activity as stated in the country’s vision for 2020. This finding is also consistent with the need for good qualifications to apply for dental education in Malaysia. However, high entrance criteria are the norm for dentistry, particularly in high-income countries [41]. While parents with higher education also expect good academic achievement in their children to facilitate better career choices [42], education is perceived as a valuable route to social advancement [43], and this may be an important driver in Malaysia during a period of economic advancement and development in the global arena [44].

A second important finding of this study was that students’ motivation varied by students’ and school characteristics. Ethnicity and school type were particularly important in this regard. Family and friends were less important (lower scores) among Malay students compared with those from other ethnic groups. Evidence from Australia and New Zealand suggests that family and friends play an important role in encouraging Asian students to study dentistry [16], and in the Middle East, this is most notable among females [21]. This could be explained by the fact that students from minority groups seek advice from their social circle regularly [45]. It would be interesting to explore this finding in further studies by including newly formed private schools, largely serving minority ethnic groups, which did not have any final-year students at the time of this study. We also found that students in private schools had higher scores on healthcare and people than those in public schools, which challenges the common assumption that students from private schools are more motivated to study dentistry for financial benefit [26]. Given the low response from private schools, further research is required to understand if this finding reflects actual views of students in private schools or some environmental factors (admissions criteria or school philosophy).

Finally, the present findings provide the first report on motivation to study dentistry at the country level using Malaysia as a middle-income country. As all dental schools mature and the dental profession expands, it will be important to evaluate the impact of additional students’ (such as high school experiences and parental occupation) and school characteristics. Our study provides important baseline data for Malaysia and East Asia. By understanding students’ motivation, educators can reflect and improve their recruitment strategies for dental schools. Furthermore, health planners and providers may be better informed to shape health policy in order to recruit and retain new graduates in the health systems, for the benefit of the patients and the public.

## Conclusion

This study confirms that academic factors were the dominant motivation to study dentistry among these Malaysian students. There were some variations in motivation by ethnicity and school sector, but not by age, sex or family income or whether the school was established or new.
